# 3D Printing and Blue Sustainability: Taking Advantage of Process-Induced Defects for the Metallic Ion Removal from Water

**DOI:** 10.3390/polym16141992

**Published:** 2024-07-11

**Authors:** Akel F. Kanaan, Ana P. Piedade

**Affiliations:** 1 Federal University of Paraná, Department of Chemical Engineering, Curitiba 82590-300, PR, Brazil; akelkanaan@ufpr.br; 2 University of Coimbra, CEMMPRE, Department of Mechanical Engineering, 3030-788 Coimbra, Portugal

**Keywords:** sustainability, wastewater treatment, additive manufacturing, porous morphology, metallic ion removal, LAY-FOMM^®^60

## Abstract

Additive manufacturing (AM), commonly known as 3D printing, allows for the manufacturing of complex systems that are not possible using traditional manufacturing methods. Nevertheless, some disadvantages are attributed to AM technologies. One of the most often referred to is the defects of the produced components, particularly the porosity. One approach to solving this problem is to consider it as a non-problem, i.e., taking advantage of the defects. Commercially, LAY-FOMM^®^60 polymer was successfully used in AM through a material extrusion process. This filament is a blend of two polymers, one of them soluble in water, allowing, after its removal from the printed components, the increase in porosity. The defects produced were exploited to evaluate the metallic ion removal capacity of manufactured components using non-potable tap water. Two experimental setups, continuous and ultrasound-assisted methods, were compared, concerning their water cleaning capacity. Results revealed that continuous setup presented the highest metallic ion removal capacity (>80%) for the following three studied metallic ions: iron, copper, and zinc. High water swelling capacity (~80%) and the increase in porosity of 3D-printed parts played a significant role in the ion sorption capacity. The developed strategy could be considered a custom and affordable alternative to designing complex filtration/separation systems for environmental and wastewater treatment applications.

## 1. Introduction

According to the world population prospects of the United Nations, the world population is projected to reach 10.9 billion in 2100, an increase of ~1.42-fold compared to 7.7 billion in 2019 [1,2]. This significant increase in global population density is expected to promote greater changes in consumption patterns, thus leading to higher goods consumption, water/air pollution generation, and scarcity of natural resources. Consequently, higher pressure on the environment is expected, overwhelmingly increasing energy, food, and water demands [1,3].

Global water shortage (also called water stress) is currently one of the main risk topics, according to the World Economic Forum [4]. Water streams are being contaminated with more significant concentrations of pesticides, surfactants, personal care products, and pharmaceutical/industrial waste, including organic compounds, dyes, and metals. Classical wastewater treatment technologies, such as physical (adsorption, coagulation/flocculation, filtration), chemical (oxidation and ozonation), and biological (enzymatic and microorganisms) technologies, are employed to generate safe, clean water [5]. Concerning the development of materials, their application in the problems of water contamination with metals and radionuclides can be used as an example, due to the impact of these substances on all living organisms. It is of the most urgency to develop/improve materials that can efficiently contribute to the removal of such contaminants, as reported in two recent reviews [6,7]. Moreover, these materials themselves cannot be contaminants or use contamination procedures/reagents/radionuclides in their preparation, as was the case at the end of last century [8,9,10].

In a broader application, novel and interesting wastewater treatment strategies to enhance clean water production have been reported, including solar energy-based [11], nanotechnology-based [12,13,14], and artificial intelligence (AI)-based [15] methods. Among the novel strategies, additive manufacturing (AM)-based wastewater treatment has received significant attention due to its affordability, complex and diverse structure manufacture capability, scalability, automation, and low waste production [16,17,18,19,20,21]. AM-based wastewater treatment methods can be regarded as an interesting alternative to conventional ones due to their capability to produce structures (filters, membranes, adsorbents) with precise control of fabrication, customizable and intricate design, complex shape, and cost-effectiveness. However, AM presents some general drawbacks, namely low resolution/accuracy (micrometric scale threshold), slow printing speed, limited material array [18], and high-energy consumption [22].

AM (commonly referred to as 3D printing (3DP)) comprises the fabrication of parts, components, and devices in a layer-by-layer disposition from a file previously designed using computer-aided design (CAD) or computed tomography (CT) data [23,24]. There are several 3DP processes employed in the design of systems for environmental applications, namely powder bed fusion, binder jetting, material jetting, vat photopolymerization, and material extrusion. The material extrusion process, specifically the fused filament fabrication (FFF) technology, is the most commonly utilized due to its affordability, availability, and low waste production [18,19]. Three-dimensionally printed systems have been explored for several water treatment applications, including fouling reduction, oil/water separation/skimming, water splitting (steam generation by solar energy absorption), water desalination, organic contaminants, and heavy metal ion removal [17,18,19,20,21]. The design of contaminant removal materials (adsorbent) through 3DP technology allows the possibility to tune its surface area, flow properties, geometry, hierarchical roughness, and mechanical properties, which significantly enhances/tunes media-adsorbent interactions. Different polymeric materials were studied in the design of 3D-printed systems for filters, membranes, and adsorbents. They include alginate [25,26,27], chitosan [28,29], cellulose [30], gelatin [31], poly(ethylene glycol) (PEG) [32], poly(dimethyl siloxane) (PDMS) [33], poly(lactic acid) (PLA) [34,35], polypropylene (PP) [36], and poly(acrylonitrile-butadiene-styrene) (ABS) [37]. LAY-FOMM^®^ is a poorly explored poly(vinyl alcohol) (PVA)/poly(urethane) (PU) blend material for water treatment/environmental applications. It has been employed for the development of several platforms for different applications, namely tissue engineering scaffolds [38], human plasma sorbents [39], stretchable textiles [40], cartilage replacements [41], glimepiride extraction [42], and rhodamine B removal [43]. Its porosity, flexibility, amphiphilic nature, and water uptake capacity make it exciting for potential water treatment applications as a custom-shaped sorbent for ion/molecule removal.

This work aims to evaluate the metallic ion removal capacity of 3D-printed LAY-FOMM^®^ 60 sorbents from water media comprising a complex mixture of electrolytes. In order to accomplish this goal, the ion sorption efficiency of 3D-printed sorbents was evaluated against two different experimental approaches, namely continuous column and ultrasound-assisted methods. The influence of the experimental setup will allow us to evaluate if each studied methodology induces ion removal selectivity, different ion removal capacities using different media samples’ contact area interactions, and the presence/absence of a mechanical stimulus. Moreover, the processed, induced defects (at a macro- and microscale) can be repeatable, if some of the printing parameters are kept constant, namely layer high, printing path, orientation, and infill percentage and geometry. Finally, this work demonstrates a simple and efficient strategy to develop mechanically stable 3D-printed porous sorbents with a modular metallic ion removal capacity, according to the type of experimental setup utilized.

## 2. Materials and Methods

### 2.1. Materials

Commercially available LAY-FOMM^®^ 60 (FOMM) filaments (diameter of 1.75 ± 0.03 mm) were purchased from Filament2Print^®^ (Nígran, Spain). The characterization of the filaments, and some of the additively manufactured specimens, was conducted on pristine and washed samples. In this case, the samples were washed in deionized water (500 mL, replaced three times a day for up to seven days at room temperature, 23 °C, with constant stirring at 100 rpm) to remove their PVA content.

### 2.2. AM of FOMM

Pyramidal FOMM samples (20 mm × 20 mm × 20 mm) were printed using FFF FlashForge^TM^ Creator 3 equipment (BeeVeryCreative, Ílhavo, Portugal) with a 0.4 mm nozzle diameter, using the following printing parameters: nozzle and bed temperature of 230 °C and 110 °C, respectively; printing speed of 25 mm/s; triangular infill pattern with 50% density; layer height of 0.2 mm; number of shells of 2; and raft enabled. The geometry, infill pattern, and density of the printed samples were chosen according to previous exploratory/preliminary studies, as briefly discussed in the results section.

### 2.3. Characterization of FOMM

#### 2.3.1. Chemical Analysis

The chemical functional groups of FOMM as-printed and washed samples were characterized via attenuated total reflection Fourier transform infrared (FTIR-ATR) spectroscopy. Spectra were acquired using Bruker, Alpha II equipment (Leipzig, Germany) with accumulation of 24 scans, 4 cm^−1^ resolution, and between 4000–400 cm^−1^ of wavenumber. Resulting spectra were analyzed using the OPUS 4.0 software (Bruker).

#### 2.3.2. Morphological Analysis

The polymeric materials were dried (50 °C for at least 48 h) before experiments. The morphological dissimilarities of FOMM (before and after the washing procedure) were observed via scanning electron microscopy (SEM). Measurements were carried out using a ZEISS^®^ MERLINTM 61-50 microscope (Carl Zeiss; Oberkochen, Germany) at 2 keV. Due to the electric insulating properties of the polymeric material, the samples were gold-sputtered under an argon atmosphere with a 5 nm thick coating.

The influence of washing procedures on printed samples’ internal and external dimensions was evaluated via computed tomography (CT). Each sample was dried at 50 °C for at least 48 h prior to experiments. The scans were obtained in a Metrotom 800 Scanner from Zeiss with the following parameters: 130 keV/39 W, 1900 × 1512 pixel detector, and a measurement volume and combined measurement volume of Ø100 × 150 mm and Ø110 × 220 mm, respectively. Each analysis was performed in different and representative cross-section regions of tested samples. CT images were acquired with the dedicated manufacturer software.

#### 2.3.3. Porosity Characterization

Mercury porosimetry was used to determine pores and size distribution of the printed specimens before and after PVA removal, using Auto Pore IV 3500 equipment from Micromeritics.

The Washburn equation (Equation (1)) determines the relation between the pore size and the applied pressure

PL − PG = 4σ cos θ/DP(1)

where PL is the pressure of the liquid, PG is the pressure of the gas (it is considered zero when the technique is performed under vacuum), σ is the surface tension of the liquid, θ is the contact angle of intrusion liquid, and DP is the pore diameter.

The samples (1g) were subjected to vacuum, and the sample holder was filled with Hg until it reached a pressure of 3.4 kPa. For the low pressure analysis (larger pores), increments were made until it reached a pressure of 138 kPa. For high pressure measurements (smaller pores), increments were made until it reached a pressure of 228 MPa. During the filler procedure, the bulk density is measured, and at the end of the higher pressure measurements, the apparent (skeletal) density is obtained. The quotient between (apparent-bulk) and apparent determines the percentage of porosity. The experiments were performed in triplicate.

#### 2.3.4. Thermal Characterization

The thermal stability of FOMM filaments was evaluated using a thermogravimetric analyzer (TA Instruments, Q500, New Castle, DE, USA). Samples (±10 mg) were characterized between 25 °C and 600 °C, at a heating rate of 10 °C/min under a nitrogen atmosphere (50 mL/min of flow rate). The filaments, printed samples, and printed samples after washing were also characterized by differential scanning calorimetry (DSC-200 F3 Maia^®^, NETZSCH, Porto, Portugal), applying consecutive heating cycles of heating (from 40 °C up to 150 °C), cooling (from 150 °C up to −80 °C), and reheating (from −80 °C up to 300 °C), at a heating rate of 10 °C/min under nitrogen atmosphere (50 mL/min).

#### 2.3.5. Swelling

The water sorption capacity (WSC) of the AM samples (before and after washing procedures) was evaluated in bidistilled water at room temperature (~23 °C). Previously dried samples (50 °C for 48 h) were weighted and immersed in 15 mL of bidistilled water. At predetermined time intervals, samples were weighted (after removing the excess surface water with filter paper) and immersed again in the corresponding medium. The WSC of printed samples was calculated as the ratio between the weight of water absorbed at time, t, and the initial dried weight (before contact at t = 0 h). The results were expressed as a g_water_/g_dried_ sample, and performed in triplicate.

#### 2.3.6. Ion Sorption

The ion removal capacity of 3D-printed samples was evaluated by the direct contact with a complex sample of “real” water. The liquid was collected from the water tap system of the garden (pH ≈ 6.0) and used without any treatment. Measurements were carried out at room temperature (~23 °C), comprising the following two different experimental setups: (1) the continuous and (2) ultrasounds-assisted methods. The continuous method consists of a sample-loaded separation column connected to a continuous flow (top to bottom oriented in a closed loop) of electrolyte solution provided by a peristaltic pump (10 mL/min) under constant stirring (1000 rpm). The ultrasound-assisted method included immersing the samples in electrolyte media and submitting them to a constant mechanical stimulus (sonication). For comparison purposes, each system comprised four previously dried samples (50 °C for 24 h) and a fixed total volume of electrolyte solution (400 mL). Moreover, measurements were carried out at predetermined time intervals of 8 h/day for eight days. Finally, the electrolyte solution was sealed with Parafilm® (Amcor, Zürich, Switzerland) in both experimental approaches to prevent liquid evaporation during measurements. Experiments were carried out in quadruplicate.

Qualitative and quantitative analysis of the electrolyte solutions and 3D-printed samples (before and after ion sorption experiments) were performed using X-ray fluorescence (XRF) and flame atomic absorption spectrometry (FAAb) techniques. Regarding XRF experiments, aliquots (10 mL) of each tested solution and two samples were analyzed in a WD-FRX AxiosmAX 4 kW with a rhodium ampoule. Semi-quantitative chemical analysis was carried out using Omnian 1.0 software (Malvern Panalytical, Almelo, The Netherlands). In the FAAb analysis, measurements in the electrolyte solutions were performed using a Perkin Elmer Atomic Absorption Spectrometer 3300 (SpectraLab Scientific Inc., Markham, ON, Canada) with an acetylene/air flame. Copper (Cu), Iron (Fe), and Zinc (Zn) were analyzed using a Cathodeon lamp (Mitorika Co., Ltd., Mito-shi, Japan) at 324.8, 248.3, and 213.9 nm, respectively. The calibration curve was tested with a control ampoule (RTC). The percentage of ion removal capacity of the samples was calculated according to the following Equation (Equation (2)):

(2) $$Ion\;removal\;capacity\;\left(\%\right)=100-\left(\frac{{\left[ion\;in\;solution\right]}_{t>0}}{{\left[ion\;in\;solution\right]}_{t=0}}\right)\times \;100$$

#### 2.3.7. Statistical Analysis

The statistical significance of the obtained data was evaluated by the one-way ANOVA test. Statistical significance was attributed to *p*-values < 0.05.

## 3. Results and Discussion

### 3.1. Exploratory/Preliminary Studies

Prior to ion sorption experiments, our research group investigated the separation capacity of 3D-printed FOMM samples against oil/water emulsions (25:75). We hypothesized that different geometries (semi-spherical and pyramidal) and hierarchical surface roughness (induced by different layer heights during the printing process) [44] would prompt the “lotus effect” [45,46,47]. Therefore, the presence of hierarchical roughness (microscale and macroscale) would reduce the contact area between the droplet and the surface of the samples by creating localized “air pockets” on the valleys of its rough surface, inducing different surface energies. Finally, the change in surface energy would prompt water repellence and enhance oil adsorption/adhesion, resulting in oil from water separation. Nevertheless, during emulsion separation/ultrasound-assisted washing cycles, it was observed that the color of FOMM samples permanently shifted from white to a peach/copper-like tone. This result suggests that 3D-printed FOMM samples could naturally adsorb/remove ions from the aqueous media. Thus, we speculated that the printed-induced hierarchical surface comprised different surface energies, which, combined with its water sorption capacity, could synergistically work as an advanced sorbent. This sorbent capacity could be potentially enhanced by its surface hierarchical roughness and water uptake capacity. Consequently, this paved the path to investigate the metallic ion sorption capacity of 3D-printed FOMM samples, as presented and further discussed in the following sections of this work.

The effect of the geometry of the printed parts was evaluated via its water contact angle. Preliminary results indicated that there was a significant difference (*p*-value > 0.05) between the semi-spherical and pyramidal geometries for the same 3D printing resolution, by FFF technology. Additionally, the influence of the infill density (25 and 50%) and pattern (triangular and hexagonal/honeycomb) was also evaluated. The parts printed with 25% of infill density showed lower mechanical stability, especially after washing procedures. When considering the influence of the infill pattern, our previous surface contact angle studies revealed no significant differences between tested infill patterns (*p*-value > 0.05), as expected. In fact, the liquids are going to contact the outer surface of the parts, not the inner part. As a result, only pyramidal-shaped samples with 50% of infill density were considered throughout this work. The justification of a triangular-shaped infill over a hexagonal/honeycomb one was merely arbitrary.

### 3.2. Chemical Characterization

**Figure 1:**
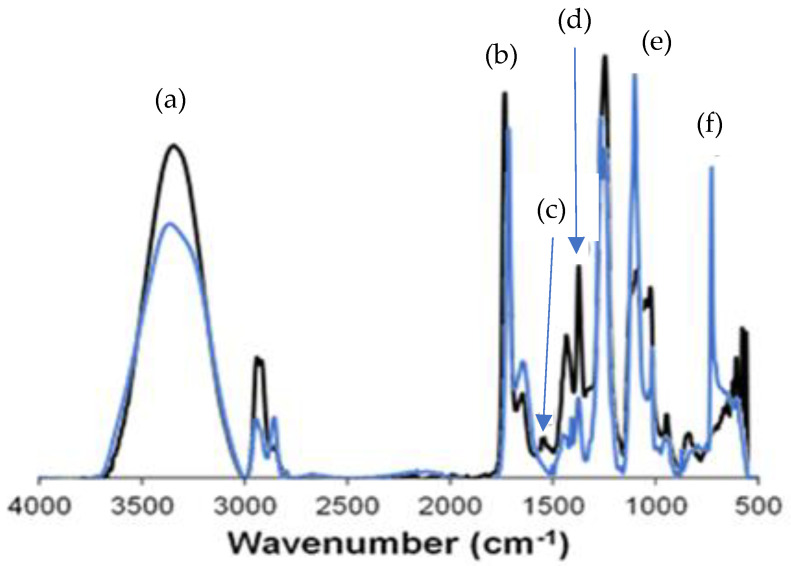
FTIR-ATR spectra of FOMM samples before (▬) and after (▬) washing procedures at room temperature (~23 °C).

LAY-FOMM^®^ 60 filaments were utilized as received, without further modification. Since it is a relatively new commercial material, a lack of information regarding its chemical composition is available in the literature. Thus, it is reported that FOMM thermoplastics comprise a PU/PVA blend [48,49]. For this reason, FTIR measurements were performed in FOMM samples before and after washing procedures (Figure 1). Obtained spectra revealed characteristic peaks assigned to PU, namely O-H vibration (at 3500–3000 cm^−1^) (a), amide I (C=O stretching at 1715–1650 cm^−1^) (b) [39], amide II (N-H bending at 1547 cm^−1)^ (c) [50], and amide III (C-N wagging at 1377 cm^−1^) (d).

The effect of the washing procedure on the chemical composition of tested samples was also analyzed by FTIR analysis. It is possible to observe that washed samples presented a lower peak intensity at 3500–3000 cm^−1^ (O-H vibration) and 1715–1650 cm^−1^ (C=O stretching) ranges by comparing the results from non-washed ones. This behavior is attributed to the removal of its PVA content (~15%wt) during washing procedures [38,48]. It also reveals PU-assigned peaks with higher intensities at 1100 cm^−1^ (e) and 726 cm^−1^ (f), attributed to C-O-C stretching vibration and =CH out of plane, respectively. This result can be explained due to the absence of the overlap of PVA characteristic peaks in washed FOMM samples, as expected.

### 3.3. Morphological Characterization

**Figure 2:**
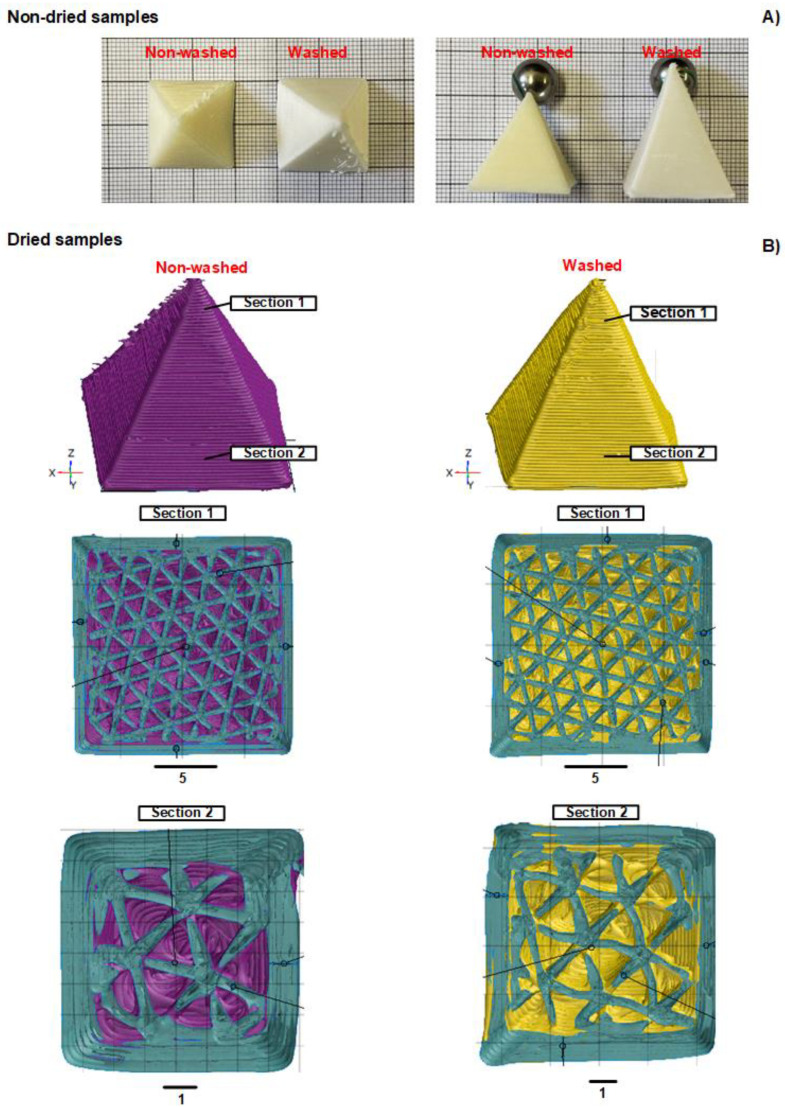
Effect of washing and drying step on the dimensions of 3D-printed FOMM samples. (**A**) Non-dried samples and (**B**) CT scans (at different cross-section heights) of dried materials. The base and height values are given by the graph paper in (**A**) with a bold square with 10 mm size.

The influence of the PVA leeching during washing and drying procedures on the morphology of 3D-printed FOMM samples was analyzed, and results are presented in Figure 2. Regarding non-dried samples, it is possible to observe that the washing step significantly influenced their dimensions (Figure 2A). Washed samples demonstrated an augment in the size of ~1.5-fold (in x, y, and z directions) when compared to non-washed ones. This elastic behavior is justified by the water uptake capacity of FOMM samples, which will be further discussed in detail in the following sections. Moreover, a change in sample coloration was also observed before and after washing procedures at room temperature (~23 °C). Washed samples presented a whiter color when compared to non-washed ones, which also indicates the leeching of their PVA content. This result agrees with the FTIR-ATR evaluation, as previously discussed (Figure 1).

In order to scrutinize the effect of the washing step on the geometry of processed materials, CT analyses were also conducted for dried samples (Figure 2B). In general, obtained tomograms, in different tested cross-sections, revealed that the size and geometry of printed samples were similar, independent of the washing procedure (19.28 mm × 19.42 mm × 19.23 mm and 19.41 mm × 19.43 mm × 20.03 mm for non-washed and dried washed materials, respectively). This result suggests that the influence of the washing step on dried samples’ dimensions was not significant (*p*-value > 0.05), considering the established geometrical data input during the 3D printing process (e.g., 20 mm × 20 mm × 20 mm). After drying, washed samples decreased their volume due to water evaporation, which justifies the observed results. CT scans also illustrated that washed samples maintained their structural integrity, with defined shape and a hierarchical surface throughout analyzed cross-section areas.

### 3.4. Thermal Characterization

**Figure 3:**
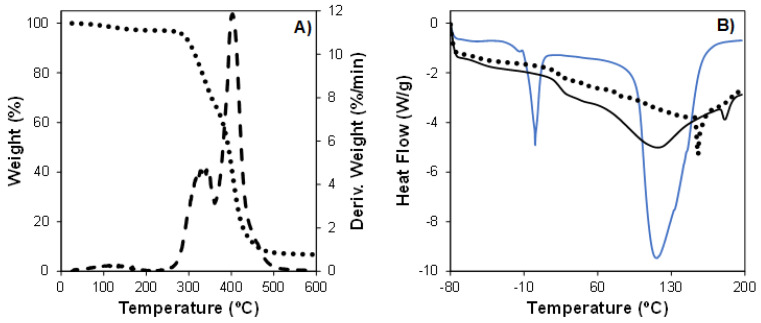
(**A**) TGA profile and differential thermogram (DTG) (dashed line) and (**B**) DSC thermograms (exothermal events oriented up) of LAY-FOMM^®^ 60 filaments (dotted line) and printed (full line) samples before (▬) and after (▬) washing procedures [41].

Since FFF is a thermal-dependent technology, the thermal properties of FOMM were evaluated using TGA and DSC characterization (Figure 3). The thermal stability of FOMM filaments (as-received) was investigated by TGA experiments (Figure 3A) to elucidate the temperatures at which the polymeric material could be processed without degradation.

In Figure 3A, it is possible to observe that FOMM filaments presented three thermal events at 100–150 °C, 326.9 °C, and 402.9 °C [41]. The first stage (weight loss of 2.9%) is attributed to water evaporation during heating. The temperature at which the filament’s thermal degradation initiates (T_onset_) was observed at 295.2 °C, which is similar to starting decomposition temperatures of polyurethane [51]. The second thermal event (at 326.9 °C) is related to the thermal decomposition of PVA present in FOMM [52]. Moreover, this event is associated with the thermal decomposition of diisocyanate into isocyanate (from the PU polymeric network) [53]. The third thermal event (at 402.9 °C) is related to the maximal thermal degradation (T_max_) of PU in the PU/PVA blend (FOMM). The maximum weight loss of tested samples was ~93.1% at 600 °C. Finally, this result allowed us to infer that printing temperature (T_printing_, 230 °C) did not induce any thermal degradation of the FOMM polymeric blend (T_printing_ < T_onset_).

As the final metallic ion sorption tests were conducted into the washed printed FOMM printed specimens, it was necessary to compare the characteristic temperatures of the as-received material (filament), with the printed specimens (as-printed), and with the washed printed specimen (without the PVA). The study was conducted using DSC, and the results are presented in Figure 3B. The analysis of this figure allows us to observe distinct endothermic events according to each type of tested sample. Unwashed printed samples presented four endothermic thermal events, as follows: −52.7 °C, 23.4 °C, 116.7 °C, and 181.4 °C. The former two events were attributed to the glass transition temperature (T_g_) of PU and PVA segments in the FOMM blend [41,54]. The latter endothermic peaks (at 116.7 °C and 181.4 °C) were associated with the melting temperature (T_m_) of PU and PVA constituents in FOMM materials (at 116.7 °C and 181.4 °C, respectively) [55]. It is important to notice that Tm values observed for the unwashed printed samples shifted (decrease of 38.7 °C), compared to unwashed FOMM filaments [41,56]. Moreover, this region was broader in the printed samples than in the filaments. This behavior suggests that a flow-oriented anisotropic molecular organization (drawing effect) took place during the extrusion printing process, resulting in a higher percentage of crystalline domain [57,58]. The effect of the washing procedure on the thermal properties of 3D-printed samples was also evaluated. It is clear that the removal of PVA significantly affected the profile of the obtained thermograms. In this case, only two well-defined endothermic events were present for washed printed samples. Endothermic peaks were observed at 0.6 °C and 116.35 °C, corresponding to PU’s T_g_ and T_m_, respectively. This result demonstrated that thermal events related to PVA were absent for washed samples compared to those observed for unwashed 3D-printed materials, as expected. Moreover, the increase in T_g_ values (increase of ~52 °C) could be attributed to the removal of PVA. The result was the decrease in the hygroscopicity of the material, thus reducing its moisture content, which decreased segmental motion of polymeric chains, ultimately increasing T_g_.

### 3.5. Water Swelling Capacity (WSC)

**Figure 4:**
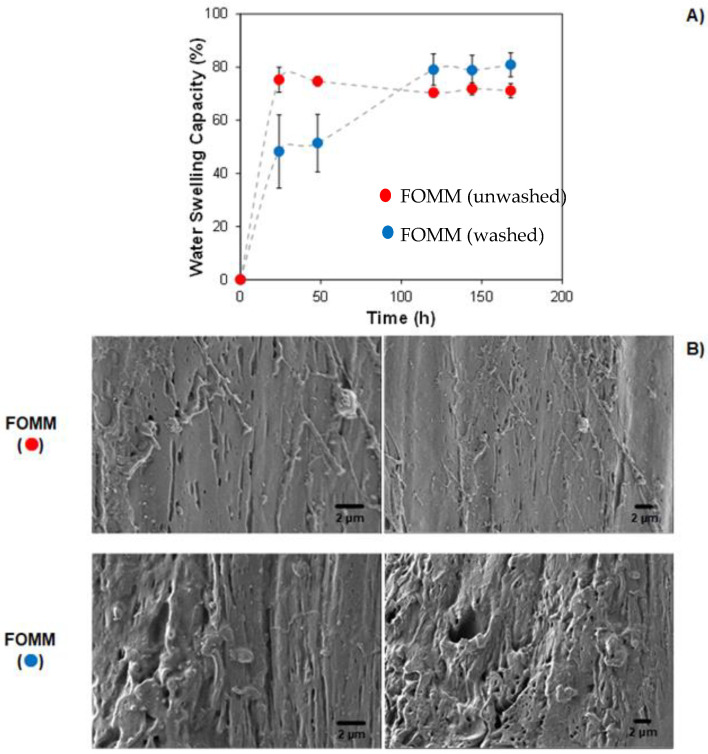
Water swelling capacity of 3D-printed FOMM samples (**A**) and cross-section SEM micrographs (**B**) before (∙) and after (∙) washing procedure [41].

The water uptake capacity of a given material is an important parameter when wastewater treatment applications are envisaged. Therefore, the WSC of 3D-printed FOMM samples was analyzed in bidistilled water at room temperature (~23 °C). In general, the results demonstrate a hydrophilic behavior of FOMM samples, which presented an average water sorption capacity of at least 70 wt.% (Figure 4A).

When comparing the effect of the washing procedure (prior to swelling experiments) on WSC, it can be noticed that the presence of PVA influenced the swelling kinetics, especially at t ≤ 50 h. The hydrophilic nature of PVA promoted higher (~1.5 × higher) water molecule diffusion towards the printed structure, augmenting its water sorption capacity and resulting in greater WSC when compared to washed samples. Moreover, both samples reached a water sorption plateau (WSC equilibrium) at t > 100 h, and the elastic response of tested materials mainly defines its water uptake threshold. Nevertheless, washed samples demonstrated a higher (*p*-value < 0.05) WSC of 79.6 ± 1.1% compared to unwashed ones. This result might be explained due to the porosity increase in washed FOMM samples (Figure 4B). It can be observed in SEM micrographs that the removal of PVA acted as a porogen agent, thus prompting the formation of cavities (free space) during its dissolution in water.

The quantification of the pores, and size distribution were made by mercury intrusion, and the average results are summarized in Table 1.

As expected, after PVA removal, the printed samples present 60.4% of increased porosity and a 61.3% increase in the median pore diameter (in volume). The removal of the PVA is responsible for increasing the porosity values. Consequently, in the studied polymeric blend, the porosity percentage and the pore diameter can be tailored by changing the percentage of PVA and the distribution of PVA in the blend, respectively. A more homogeneous distribution will imply smaller pore diameters regularly distributed in the samples, while a heterogeneous distribution will give rise to higher pore diameters irregularly distributed in the printed specimens.

The presence of pores throughout the FOMM structure, which increased the contact area, prompted higher water penetration by diffusion and capillarity mechanisms, thus resulting in greater WSC [59]. The average WSC values at equilibrium, herein obtained for the printed samples (independent of been washed or not), were substantially greater than those obtained for PU-based systems [60,61]. This result can be explained due to the presence of pores and the different chemical nature of samples, which hampers a fair comparison of results. Finally, all samples presented mechanical and geometrical integrity, with defined size, shape, and hierarchical roughness for at least 6 months in contact with bidistilled water at room temperature (~23 °C).

### 3.6. Metallic Ion Removal Capacity of 3D-Printed FOMM Sorbents

**Figure 5:**
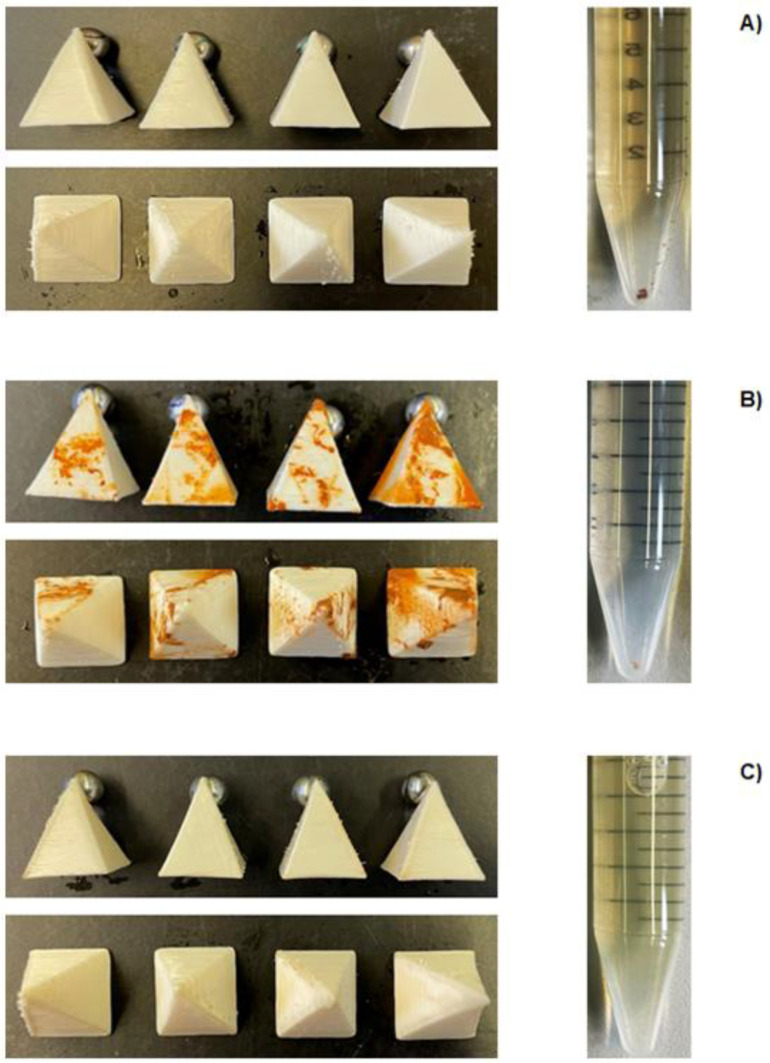
Illustration of 3D-printed FOMM sorbents and electrolyte aqueous media (**A**) before (t = 0 h) and after (t = 68 h) ion sorption experiments carried out in (**B**) continuous and (**C**) ultrasound-assisted setups.

The ion sorption capacity of 3D-printed sorbents was evaluated in function of two different experimental setups (continuous and ultrasound-assisted methods) for samples in contact with tap water medium (as model electrolyte solution). Figure 5 illustrates the visual aspect of all tested samples and electrolyte solutions, before and after ion removal experiments, for both experimental setups employed. In general, the type of experimental setup exerted an influence on the ion removal capacity of each tested sample. It was possible to verify an alteration in the coloration of samples (from white to orange/pale yellow) and electrolyte media when compared to the control (Figure 5A). This color alteration indicates that the samples were able to remove (adsorb) the ions naturally present in the electrolyte aqueous media (orange color probably due to the presence of iron oxide, Figure 5A).

It is important to note that the adsorption process is time-dependent [62]; thus, experiments were carried out until any color alteration of the tested sample or electrolyte media was visually noticed (sorption equilibrium) (t = 68 h). Regarding continuous setup (Figure 5B), samples visually presented a higher ion removal capacity when compared to the ones from the ultrasound-assisted method (Figure 5C).

FOMM adsorbents from the continuous setup presented a strong orange color surface distribution, which suggests a greater ion sorption capacity. The surface color distribution was heterogeneous among the samples, due to the organization of the samples inside the separation column. This cascade-like organization prompts the ion removal of the samples positioned on the top of the separation column (higher contact area), when compared to the ones located at the bottom. An ion gradient concentration occurs, with it being higher at the top of the column and lower at the bottom, at a given time interval. Due to the experimental setup, samples located at the top would first adsorb the metallic ions and, consequently, the concentration of these species is lower when the water reaches the samples located at the bottom. As a result, it renders a heterogeneous ion removal (clearly noticed by uneven color distribution), as previously mentioned.

Regarding the samples from the ultrasound-assisted setup (Figure 5C), these samples visually presented a lower ion sorption capacity (if compared to those from the continuous method) due to the lighter coloration (pale yellow) of the samples. Nevertheless, the presence of mechanical stimuli (ultrasound) led to a homogenous ion sorption distribution along the 3D-printed sorbents. This result can be explained due to the higher surface contact area of the samples with the electrolyte media. The ion sorption capacity behind all tested samples (independently of studied experimental setup) is given by diffusion mechanisms. When samples are in contact with electrolyte solutions, an ion concentration gradient takes place (greater in the aqueous media), leading to diffusion of ions towards FOMM sorbents. Additionally, the hydrophilic nature of 3D-printed samples, as well as its microporosity (as observed in Figure 4A and Figure 4B, respectively) and “macroporosity” (due to infill of 50%), have favored ion removal by diffusion mechanism. Nevertheless, other PU-based ion removal systems have mentioned that metallic ion sorption can also be given through the chelation mechanism [60]. Since FOMM is a relatively new and poorly explored material, more studies need to be carried out in order to fully determine the chemical nature/structure of this polymer, and, thus, define its specific metallic ion sorption mechanism. Finally, by visually comparing the coloration of obtained water after ion sorption measurements, the color intensity varies, as follows: continuous < ultrasound-assisted < control (electrolyte solution before experiments at t = 0 h). This color intensity trend follows an inverse pattern for FOMM samples after ion sorption experiments, as expected.

**Figure 6:**
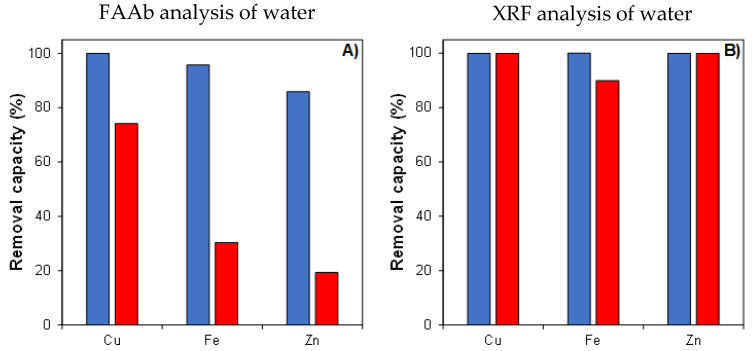
Influence of experimental setup on the ion removal capacity (quantification in the water, after ion removal), evaluated by FAAb (**A**) and XRF (**B**). The symbols were coded as the continuous (☐) and ultrasound-assisted (☐) methods.

Semi-quantitative and qualitative analyses (FAAb and XRF in Figure 6A and Figure 6B, respectively) were carried out in water after the experiments to determine the percentage of metallic ions still present in the water; the results were then translated to ion removal capacity, with the removal of ions presented in the aqueous media. The atomic adsorption results for the water before the experiments (at t = 0 h) revealed the presence of different ions, namely iron [13.08 mg/L], zinc [4.58 mg/L], and copper [0.06 mg/L].

Therefore, the presence of iron (Fe) and copper (Cu) ions in solution characterize the orange-like color of the electrolyte solutions, as previously mentioned. Moreover, the results clearly demonstrate that the type of experimental setup influenced the metallic ion removal capacity of FOMM sorbents (Figure 6A). In general, the continuous experimental approach presented a higher ion removal capacity (up to 4.4 × greater) when compared to the ultrasound-assisted setup, independent of the chemical nature of the ionic species in solution. This result is in agreement with the visual analysis described in Figure 5. A reasonable justification for this result is that the constant application of a mechanical stimulus (sonication) simultaneously induces higher sorption (by prompting the ion/sorbent interactions) and ion desorption (induced leeching/release of adsorbed ions) until reaching an equilibrium (by gradients of concentration). Conversely, the absence of sonication on samples submitted to the continuous experimental approach allowed the ions to fully permeate all samples (from top to bottom orientation). Thus, this configuration allows for greater ion retention/filtration (by tortuosity and diffusion mechanisms), resulting in a higher ion sorption removal capacity, which justifies the trend herein, presented in Figure 6A. Here, it is clear that the continuous mode was able to remove almost all of the metallic ions, when compared to the initial concentration.

Complementary to FAAb experiments, XRF analysis, also conducted after the ion sorption experiments, were carried out in the water (Figure 6B) and on pyramidal dried samples (Table 2). The quantification of the ions in the dried pyramids could not be made by FAAb because this technique cannot be used in solid samples.

Considering the electrolyte solutions, it was possible to verify the absence of the three types of ions in the continuous mode, similarly to the FAAb. However, in contrast to the FAAb results, the ultrasound-assisted method also promotes an almost perfect elimination of the metallic ions from the water. These differences might be attributed to the resolution/threshold detection of each technique, being that FAAb is more accurate in determining minor/trace elements than XRF.

In the case of results observed for dried FOMM sorbents after ion removal experiments (Table 2), they verified the presence of several ions, including those previously detected, such as Fe, Cu, and Zn, and new ones such as chlorine (Cl) and sulfur (S). The major percentage was detected for Si, which is considered a contaminant to the XRF experimental setup. By comparing the results observed for the control (washed sample in bidistilled water), it was possible to observe a significant influence of the experimental setup on ion removal, especially for Fe and Zn ions. In general, the concentration of the remaining ions (Cu, Cl, and S) was lower (up to 2.35 × inferior), due to the increase in the concentration of Zn and Fe ions (in a fixed volume of sample) for continuous and ultrasound-assisted setups. Additionally, the results demonstrated that the continuous experimental approach presented a greater Fe and Zn removal capacity (~4.4 and 2 × higher, respectively) when compared to the results observed for the ultrasound-assisted method. This result agrees with the trend observed for FAAb analysis, as previously demonstrated in Figure 6A.

## 4. Conclusions

FFF technology could be successfully employed in the development of flexible, porous, and hydrophilic FOMM sorbents. Tested materials were able to remove large quantities (>80%) of metallic ions from complex electrolyte solutions. Samples were able to adsorb several ions (e.g. Fe, Cu, and Zn) according to diffusion mechanisms. The hydrophilic nature, and the micro- and macroporosity of the printed FOMM specimens prompted an ion removal capacity of developed materials. The type of experimental setup was demonstrated to be an easy and cheap variable to control the final ion removal efficiency. The continuous setup was demonstrated to be the experimental approach with higher removal efficiency when compared to the ultrasound-assisted method. No clear evidence of metallic ion selectivity was observed during experiments, which suggests that no ionic/chemical or ion/FOMM interactions took place. This work broadened the range of applications for 3D-printed LAY-FOMM^®^ 60 materials, particularly for environmental and wastewater treatment applications. Developed sorbents could be an interesting alternative for the design of porous filters and flexible separation membranes for ion/molecule removal applications.

## Figures and Tables

**Table 1:** Quantification of the porosity characteristics in the FOMM printed sample before and after PVA removal.

	FOMM _Unwashed_	FOMM _washed_
Total pore area (m^2^/g)	8.7	11.7
Median pore diameter (volume) (μm)	30.5	49.7
Median pore diameter (area) (nm)	8.6	9.3
Bulk density (g/mL)	1.42	1.00
Apparent density (g/mL)	1.60	1.23
Porosity (%)	11.0	18.2

**Table 2:** Relevant metallic ions present in the dried FOMM pyramids after ion removal experiments, determined by XRF.

	Metallic Ions in the FOMM (%)						
Specimens	Fe	Zn	Cl	S	Cu	Si	Total
Before experiment	2.18	0.83	20.83	4.08	1.63	70.51	100
After continuous	28.67	8.33	8.85	1.83	1.21	51.12	
After ultrasound	6.53	4.11	15.72	2.90	1.03	69.70	

## Data Availability

The original contributions presented in the study are included in the article, further inquiries can be directed to the corresponding author.

## Abbreviations

3D	Tridimensional
ABS	Poly(acrylonitrile-butadiene-styrene)
AM	Additive manufacturing
AI	Artificial intelligence
CAD	Computer-aided design
CT	Computer tomography
DSC	Differential scanning calorimetry
DTG	Differential thermogram
FAAb	Flame atomic absorption spectrometry
FFF	Fused filament fabrication
FTIR	Fourier transform infrared spectroscopy
PDMS	Poly(dimethyl siloxane)
PEG	Poly(ethylene glycol)
PLA	Poly(lactic acid)
PP	Polypropylene
PU	Polyurethane
PVA	Poly(vinyl alcohol)
SEM	Scanning electron microscopy
TGA	Thermogravimetric analysis
WSC	Water sorption capacity
XRF	X-ray fluorescence
